# Canakinumab treat-to target strategies increase complete response rate in CAPS

**DOI:** 10.1186/1546-0096-13-S1-P173

**Published:** 2015-09-28

**Authors:** J Kuemmerle-Deschner, F Hofer, T Endres, B Kortus-Goetze, N Blank, E Weißbarth-Riedel, C Schuetz, T Kallinich, K Krause, C Rietschel, G Horneff, SM Benseler

**Affiliations:** 1 University Hospital Tuebingen, Department of Pediatrics, Division of Pediatric Rheumatology, Tuebingen, Germany; 2 University Medical Center, Philipps University Marburg, Department of Internal Medicine and Nephrology, Marburg, Germany; 3 University Hospital Heidelberg, Haematology, Oncology and Rheumatology, Heidelberg, Germany; 4 University Hospital Eppendorf, Pediatric Rheumatology Clinics, Hamburg, Germany; 5 University Hospital Ulm, Department of Pediatrics and Adolescents Medicine, Ulm, Germany; 6 Charite Campus Virchow, Childrens Hospital, Section Rheumatology, Berlin, Germany; 7 Charite Campus Mitte, Allergie-Centrum Charite, Department for Dermatology, Berlin, Germany; 8 Clementine Childrens Hospital, Rheumatology, Frankfurt, Germany; 9 Asklepios Klinik Sankt Augustin, Centre for Pediatric Rheumatology Sankt Augustin, Sankt Augustin, Germany; 10 University of Calgary, Rheumatology, Alberta Children's Hospital, Calgary, Canada

## Objective

Cryopyrin-associated periodic syndrome (CAPS) is a heterogeneous group of diseases characterized by excessive Interleukin-1β (IL-1 β) release resulting in severe systemic and organ inflammation. Canakinumab targets IL-1β and is approved at standard dose for children and adults with all CAPS phenotypes. Limited data are available regarding real-life effectiveness of canakinumab in patients living with CAPS. Therefore the aim of the study was to evaluate the real-life dosing practice and effectiveness of canakinumab in CAPS.

## Methods

A multi-center study of consecutive children and adults with CAPS treated with canakinumab was performed. Demographics, CAPS phenotype and disease activity, inflammatory markers and canakinumab treatment strategy were recorded. Treatment response was assessed using CAPS disease activity scores, CRP and/or SAA levels. Comparisons between age groups, CAPS phenotypes and centers were conducted.

## Results

A total of 68 CAPS patients at nine centers were included, these were 31 males and 37 females; median age was 25 years and 27 (40%) were children. All CAPS phenotypes were represented. Median follow up was 28 months. Overall, complete response (CR) was seen in 72% of CAPS patients, significantly less often in severe (14%) than in mild CAPS phenotypes (79%). Only 53% attained CR on standard dose. Dose increase was more commonly required in children (56%) than in adults (22%). Centers with treat-to-target approach achieved significantly higher CR rates (94% vs 50%).

## Conclusion

Real-life effectiveness of canakinumab in CAPS was significantly lower than in controlled trials. Treat-to-target strategies may improve the outcome of children and adults living with CAPS.

